# Determinants of epilepsy among outpatients in public health institutions of Dawo district, South West Shoa Zone, Oromia, Ethiopia, 2023: an institutional-based unmatched case-control study

**DOI:** 10.3389/fneur.2024.1449659

**Published:** 2025-01-15

**Authors:** Teshoma Alemu, Abera Cheru, Lema Daba, Takele Tiki, Meseret Ifa

**Affiliations:** ^1^Department of Public Health, Dawo District Health Office, South-West Shoa Zone, Waliso, Ethiopia; ^2^School of Environmental Health Science, College of Health and Medical Science, Haramaya University, Harar, Ethiopia; ^3^Department of Emergency and Critical Care Nursing, College of Health and Medical Sciences, Haramaya University, Harar, Ethiopia; ^4^Department of Psychiatry, College of Medicine and Health Sciences, Ambo University, Ambo, Ethiopia; ^5^Department of Public Health, College of Medicine and Health Sciences, Ambo University, Ambo, Ethiopia

**Keywords:** determinants, epilepsy, outpatient, Dawo district, Ethiopia

## Abstract

**Background:**

Globally, in ~50% of epilepsy cases, the underlying cause remains unknown, despite the fact that various disease pathways may contribute to the condition. Nearly 80% of people with epilepsy live in low- and middle-income countries and the risk of premature death in people with epilepsy is up to three times higher than that for the general population. Identifying the determinants of epilepsy is important for applying evidence-based interventions to achieve a better outcome. However, this information is scarce in a country with limited resources like Ethiopia.

**Objective:**

This study aimed to assess the determinant of epilepsy among outpatients in Dawo public health institutions, from 29 February to 15 April 2023.

**Method:**

An institution-based unmatched case-control study design was used, involving 61 cases and 122 control study subjects, who were selected using a consecutive sampling technique from public health institutions in Dawo. Data were collected using a pre-tested structured questionnaire. The data were coded, entered into EpiDATA version 3.1, and exported to SPSS version 20, for further analysis. Descriptive analysis was used to determine the percentages and frequency distributions. Logistic regression analysis was used to identify the determinants of epilepsy, and a variable with *p* < 0.05 was considered statistically significant.

**Results:**

A total of 61 cases and 120 controls were included in the study, with an overall response rate of 98.90%. The majority of participants, 38 (62.3) of the cases and 63 (52.5) of the controls, were farmers by occupation. A family history of epilepsy (AOR = 13.71 95% CI 3.030–22.006), history of febrile seizure (AOR = 14.57 95% CI 2.930–24.522), history of head injury (AOR = 6.853 95% CI 1.780–16.402), and non-use of latrine were found to be determinants of epilepsy (AOR = 0.028 95% CI 0.008–0.040).

**Conclusion and recommendations:**

The current study identified a family history of epilepsy, a history of febrile seizures, head injury, and unavailability of latrines as independent predictors of epilepsy in the study area. The information that adverse febrile seizures increase the risk of epilepsy suggests that a significant proportion of epilepsy cases in Dawo district could be prevented through improved maternal, neonatal, and child care. It is recommended that the Dawo Health Office, along with relevant stakeholders, focus on addressing this issue at various levels.

## Introduction

Epilepsy is a chronic, non-communicable brain disorder characterized by recurrent epileptic seizures caused by the abnormally rapid discharge of cerebral neurons or brain cells (1). A clinical diagnosis of epilepsy is made if there have been at least two or more unprovoked seizures separated by more than 24 h, one unprovoked seizure with a high likelihood of subsequent seizures in the next 10 years (1). However, epilepsy is clinically difficult to diagnose and needs electroencephalogram (EEG) support, which is unavailable in many centers in developing countries (2). The classification of epilepsy is complex; however, the 2017 International League Against Epilepsy (ILAE) classification system includes focal epilepsy, generalized epilepsy, combined generalized and focal epilepsy, unknown epilepsy, and also considers etiology at each stage (3).

Epilepsy determinants are conditions that are associated with an increased frequency of epilepsy and differ between childhood epilepsy and epilepsy which occurs later in life. Head injury, perinatal insults, central nervous system (CNS) infections, and febrile convulsions are some examples of factors contributing to epilepsy. Additionally, the role of genetic factors in the etiology of epilepsy is now well recognized (4). Childhood-onset epilepsy often stems from genetic defects affecting ion channels or receptors. In contrast, epilepsy emerging later in life is frequently linked to cerebrovascular diseases such as stroke, particularly in individuals aged over 65 years. Epilepsy is associated with several infections, the majority of which are preventable. Understanding these causes can assist medical professionals in making differential diagnoses and providing the most effective preventative efforts (5).

An estimated five million people worldwide are diagnosed with epilepsy each year, with 49 out of every 100,000 people affected in high-income countries. According to a recent estimate, over 2.3 million Americans, including 467,711 children aged 0–17, have epilepsy, with the disease's direct and indirect costs in the United States amounting to $15.5 billion annually. The prevalence of epilepsy was estimated to be 0.42% in the 2011 cycle of the Canadian Community Health Survey.

The Global Burden of Disease 2016 report reveals that, in terms of age-standardized DALY rates for neurological disorders, epilepsy ranked second to eighth, depending on the geographic region (6). Additionally, nearly four in five patients with epilepsy in sub-Saharan Africa experienced their first seizure attack before the age of 18 years (2).

Epilepsy affects the social, psychological, and physical aspects of patients, with significant economic implications due to healthcare needs and lost productivity. The achievement of the Sustainable Development Goals (SDGs) will not be possible without substantial investment in both physical and mental health for all individuals, including those living with epilepsy (9). Additionally, epilepsy impacts patients' ability to work. A behavioral risk factor surveillance survey across 19 states found that individuals with a history of epilepsy had a lower annual household income and were more likely to be unemployed (10).

Due to the increased risk of endemic conditions such as neurocysticercosis, tuberculosis, HIV, cerebral malaria, as well as the incidence of road traffic injuries, birth-related injuries, and variations in medical infrastructure, preventive health programs, and accessible care, the prevalence of epilepsy has reached 139 per 100,000. Approximately 80% of individuals with epilepsy live in low- and middle-income countries. The risk of dying prematurely is up to three times higher in individuals with epilepsy than in individuals without epilepsy (2, 7).

Epilepsy is a public health issue in Ethiopia, with an estimated prevalence of 5.2 per 1,000 individuals at risk and an annual incidence of 64 per 100,000 individuals in large-scale, rural, and community-based studies (7, 8). According to the findings of the current study, epilepsy is associated with significantly higher mortality, health-related and social transfer costs, home care use, and lower levels of employment. Approximately half of the adults with epilepsy have at least one other health condition; their seizures are exacerbated, and their quality of life is reduced due to psychiatric problems such as depression and anxiety (9).

Some modifiable determinants for epilepsy are CNS infections, which account for ~2%−3% of epilepsies in high-income countries and ~5% in low- and middle-income countries, Traumatic brain injury is the cause of epilepsy in 4% of cases in low -and middle-income countries and in 5% of cases in high-income countries, Stroke is also a common potentially preventable cause of epilepsy, representing 12% of epilepsies in the high-income countries and 2.7% in low-income countries. Perinatal insults generally account for 25% of epilepsy cases (9). Epilepsy is the primary serious complication of febrile seizure (11).

Poor understanding of the determinants and misconceptions about epilepsy as a form of insanity and contagious disease contribute to the disease burden. Primary prevention of epilepsy requires improving maternal healthcare and obstetrical services, communicable disease control, injury prevention, and cerebrovascular health; however, the barriers to funding epilepsy research are higher in low- and middle-income countries, where funding comes from domestic organizations and the majority of funding is directed toward communicable diseases (9).

Although several studies have shown that Ethiopia has a high burden of epilepsy, little is known about its determinants, especially in the study area, which is known to be a malaria hotspot and to have a high rate of both commercial and local alcohol consumption, both of which increase the risk of CNS infection. Therefore, this study aims to assess the determinants of epilepsy among outpatients in public health institutions in Dawo, South West Shoa Zone, Oromia region, Ethiopia.

## Methods and materials

### Study design and setting

**Figure 1:**
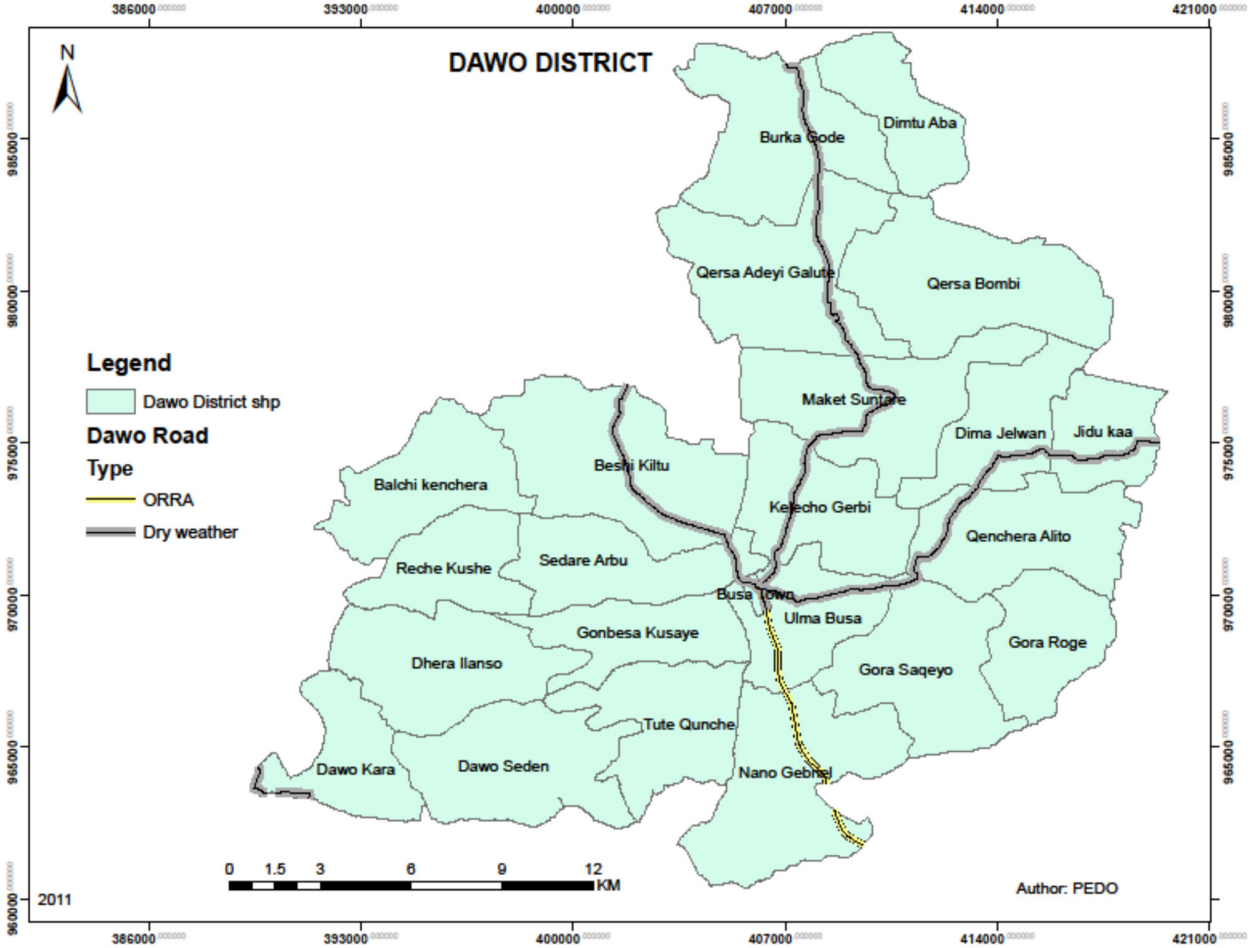
Show administrative map of Dawo district, 2023 (Source: Dawo District Land Management office).

An unmatched case-control study was conducted in public health institutions of Oromia Regional State in South West Shoa Zone Dawo district. Dawo district is one of the 11 districts in the southwest Shoa zone and is located 97 km south of Addis Ababa, the capital city of the country. It has 23 Kebeles (the smallest administrative unit): 1 urban and 22 rural. Busa is the district's capital town. In 2022, the estimated population of the district was 108,702, of which 54,384 were male and 54,318 were female. The district has four functional health centers. The research was carried out from 29 February to 15 April 2023 (Figure 1).

### Populations and eligibility

All outpatients in public health institutions in the Dawo district were the source populations, while the selected outpatients, based on the case and control definitions, were the study populations of this research. Patients who had experienced at least two unprovoked seizures in the past 2 years (epileptic clients) and were residents of the Dawo district were included in this study. Subjects with a history of isolated (single) seizures and those who were unavailable due to illness during the study period were excluded (Figure 1).

### Sample size determination

The sample size was calculated using the statistical program of the Open EPI with the double population proportions formula. A 5% level of significance (two-sided), a power of 80%, and a two-to-one allocation ratio of control to case (2:1) were assumed. To determine the sample size, the variables with the highest sample were selected. Since a family history of epilepsy resulted in the maximum sample size, it was taken to calculate the sample size. After adding 10% for the non-response rate, the final sample size was 61 cases and 122 controls, for a total of 183 study participants (Table 1).

**Table 1:** Sample size determination with different variables taken from previous studies to set sample size for a study conducted in public health institutions in Dawo district, Southwest Shoa zone, Oromia, Ethiopia, 2023.

**S. no**	**Significant determinants**	**% of cases with exposure**	**% of controls exposed**	**Power**	**AOR at 95% CI**	**Sample size**			**References**
						**Case**	**Control**	**Total**	
1	Head injury	96.5	71.4	80	11.01	28	55	83	(15)
2	Cerebral malaria	31.7	2.4	80	3.34	22	43	65	(16)
3	Family history of epilepsy	25.4	7.5	80	4.2	55	109	164	(17)
4	Febrile seizure	27.4	3.5	80	3.8	34	68	102	(13)

### Sampling technique and procedures

**Figure 2:**
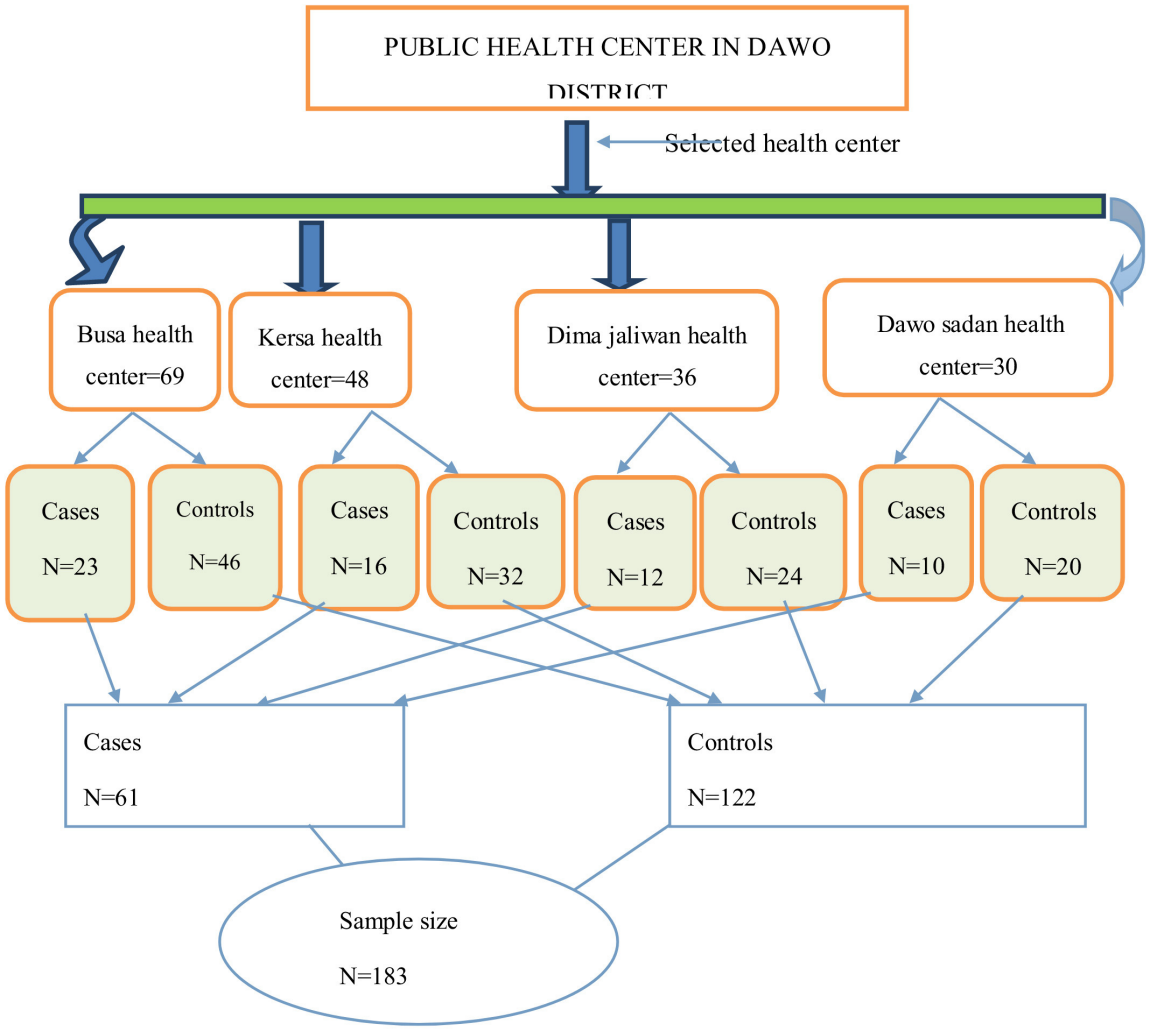
Schematic presentation to show sampling procedure for the determinants of epilepsy among outpatients in public health institutions in Dawo, 2023.

All health centers that were providing service during the study period were selected, and the calculated sample size was distributed proportionally to each selected health center based on the monthly patient flow. A consecutive sampling technique was used to select the study participants for this study until the calculated sample size was attained (Figure 2).

### Data collection tools and techniques

The data were collected using structured questionnaires, which were adapted by reviewing previous studies (1, 4, 5). The questionnaire included questions related to demographic data such as age, sex, occupation, marital status, educational status, history of seizures, and treatment. It also included detailed information on adverse events such as febrile convulsions, previous head trauma, family history of epilepsy, cerebral infection, risk factors for cysticercosis, and history of drinking alcohol before the onset of epilepsy. The data were collected by six trained data collectors.

### Study variables

Epilepsy status was the outcome variable, while socio-demographic characteristics (age, religion, sex, occupation status, ethnic group, educational status, and marital status), cerebral infection (a risk factor for cysticercosis, cerebral malaria, cerebral meningitis, and TB meningitis), drinking alcohol (everyday drinker, occasional, and none), family history of epilepsy, history of head injury, and febrile seizure were the independent variables.

### Operational definitions

**Epilepsy:** Epilepsy is defined as recurrent (two or more) epileptic seizures, unprovoked by any immediate cause (18).

**Cases:** People confirmed as living with epilepsy by witnesses and health professionals.

**Controls:** People confirmed as not having epilepsy by witnesses and health professionals.

**Febrile Seizure:** Seizures triggered by a fever of at least 100.4° F (~38°C) (6).

### Data quality control

Data collectors and supervisors were trained for 1 day. On each data collection day, the supervisors collected all the data from data collectors, and all collected data were reviewed by an investigator for completeness, accuracy, and clarity. The data collection instrument was pre-tested on 5% of the sample size at Tulu Bolo Health Center, which was not included in the final study.

### Data processing and analysis

The data were entered in the EpiDATA version 3.1 computer program to minimize data entry errors. The entered data were exported to Statistical Package for Social Sciences (SPSS) version 20 for recoding, categorizing, sorting, and further analysis. Continuous explanatory variables were categorized. Descriptive analysis was used to describe the mean with standard deviation, percentages, and number distributions of the respondents by socio-demographic characteristics and other relevant variables in the study.

Logistic regression was used to identify determinants of epilepsy. Before performing binary logistic regression analysis, collinearity diagnostics were performed. The results showed that there was no problem with multicollinearity. The goodness-of-fit of the model was checked using the Hosmer and Lemeshow test (*p* = 0.350). All explanatory variables that were associated with the outcome variable in bivariate analysis with a *p*-value of 0.25 or less were identified as candidate variables for a multivariable logistic regression model. The crude and adjusted odds ratios, together with their corresponding 95% confidence intervals, were computed. A *p*-value of < 0.05 was considered statistically significant in this study. Finally, the results were presented in text, tables, and figures.

## Results

### Socio-demographic characteristics

A total of 61 cases and 120 controls were included in the study, with an overall response rate of 98.90%. The mean age of the study participants was 31 ± 11 standard deviation. According to this study, the majority of participants, 58 (95.1%) of the cases and 83 (69.2%) of the controls, were members of the Orthodox Christian community. Concerning education status, the majority of cases had no formal education 35 (57.4%), while the majority of controls had primary education or higher 92 (76.7%). No significant differences were observed between cases and controls in terms of gender distribution: 27 (44.3%) of cases and 54 (45%) of controls were female, while 34 (55.7%) of cases and 66 (55%) of controls were male. The majority of participants, 38 (62.3%) of the cases and 63 (52.5%) of the controls, were farmers by occupation. In terms of marital status, the majority of cases were single 31 (50.8%), while the majority of controls 89 (74.2%) were married (Table 2).

**Table 2:** Socio-demographic characteristics of study participants in public health institutions in Dawo District, 2023.

**Variable**	**Category**	**Number of cases (%)**	**Number of controls (%)**
Age	0–19	16 (26.2)	14 (11.7)
	20–39	27 (44.3)	82 (68.3)
	40–59	18 (29.5)	24 (20)
Sex	Female	27 (44.3)	54 (45)
	Male	34 (55.7)	66 (55)
Educational status	No formal education	35 (57.4)	28 (23.3)
	Primary education and above	26 (42.6)	92 (76.7)
Occupational status	Farmer	38 (62.3)	63 (52.5)
	Government employee	1 (1.6)	12 (10)
	Other	22 (36.1)	45 (37.5)
Religion	Orthodox	58 (95.1)	83 (69.2)
	Protestant	1 (1.6)	33 (27.5)
	Other	2 (3.3)	4 (3.3)
Marital status	Single	31 (50.8)	30 (25)
	Married	27 (44.3)	89 (74.2)
	Divorced	3 (4.9)	1 (0.8)

### Clinical and other determinant characteristics

The study revealed that 19 (31.2%) of the cases and 6 (5%) of the controls had a family history of epilepsy. It also found that 13 (21.3%) of the cases and 7 (5.8%) of the controls had a history of febrile seizures. The study disclosed that head injury was reported by 23 of the cases (37.7%) and 15 (12.5%) of the controls. Additionally, the prevalence of cerebral malaria was reported in 9 (14.75%) of the cases and 5 (4.2%) of the controls. Cerebral meningitis was reported in 3 (4.9%) of the cases and in 5 (4.2%) of the controls. The study also revealed that 3 (4.9%) of the cases and 2 (1.7%) of the controls were everyday drinkers; 48 (78.7%) of the cases and 70 (58.3%) of the controls were drinking occasionally; and 10 (16.4%) of the cases and 48 (40%) of the controls were not drinking alcohol at all (Table 3).

**Table 3:** Clinical and other determinant characteristics of study participants in public health institutions in Dawo district, 2023.

**Variables**	**Category**	**Case no (%)**	**Control no (%)**
Family history	Yes	19 (31.2)	6 (5)
	No	42 (68.8)	114 (95)
Febrile seizure	Yes	13 (21.3)	7 (5.8)
	No	48 (78.7)	113 (94.2)
Head injury	Yes	23 (37.7)	15 (12.5)
	No	38 (62.3)	105 (87.5)
History of drinking alcohol	Every day	3 (4.9)	2 (1.7)
	Occasional	48 (78.7)	70 (58.3)
	None	10 (16.4)	48 (40)
Cerebral malaria	Yes	9 (14.75)	5 (4.2)
	No	52 (85.25)	115 (95.8)
Cerebral meningitis	Yes	3 (4.9)	4 (3.3)
	No	58 (95.1)	116 (96.7)
Latrine	Yes	6 (9.8)	94 (78.3)
	No	55 (90.2)	26 (21.7)

### Distribution of seizures at the onset

**Figure 3:**
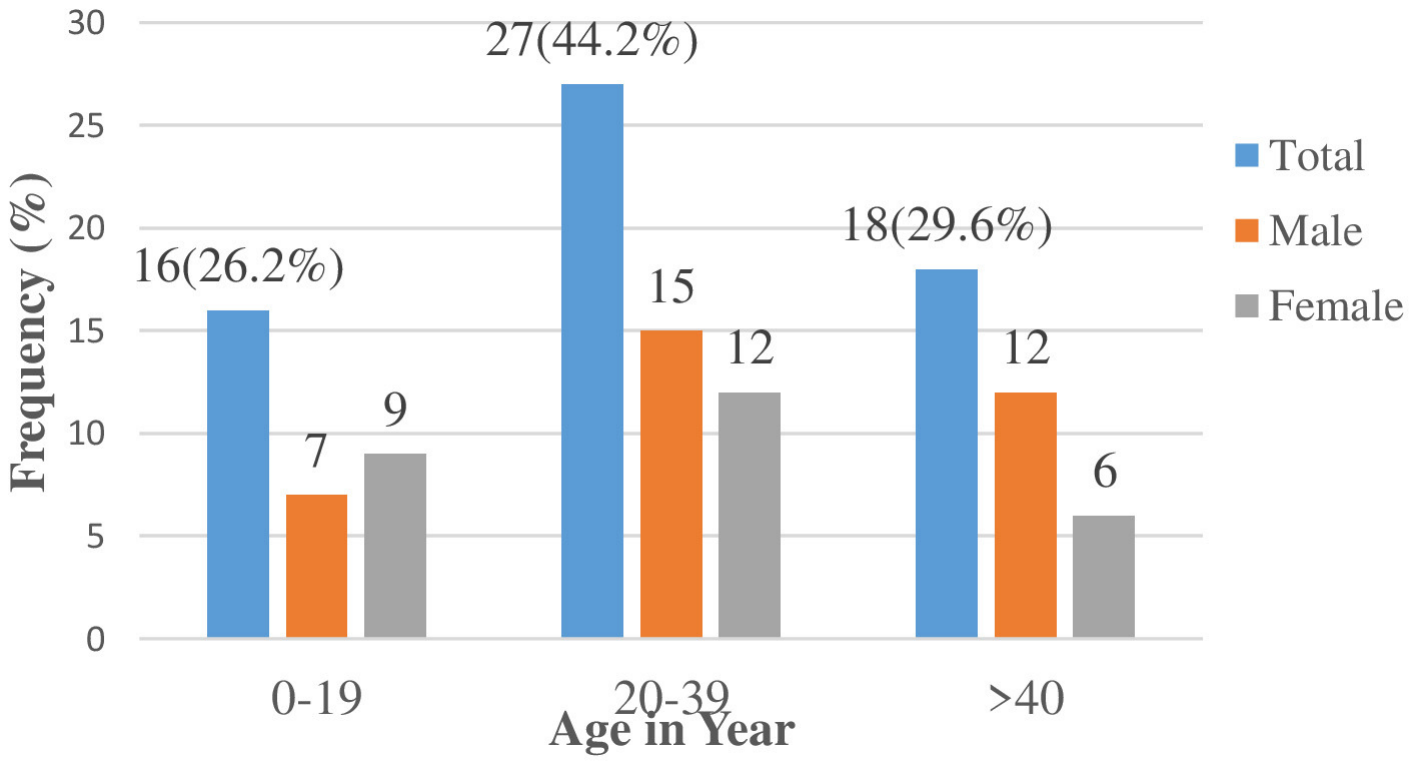
Onset of unprovoked seizures among cases in public health institutions of Dawo district 2023.

Men accounted for 55.7% of the cases, while women accounted for 44.3%. In 26.2% of the cases, the onset of epilepsy occurred within the first two decades of life, and in 73.8% in a later stage of life (Figure 3).

### Classification of seizures

Generalized seizures were encountered in 57 (93.4%) of the patients and focal seizures were in encountered 4 (6.6%). Within the group, generalized tonic-clonic seizures were the commonest type.

### Determinants of epilepsy among study participants

To investigate the association between independent variables and epilepsy, both bivariate and multivariable logistic regression analyses were employed. Variables that showed an association with the outcome variable at a *p*-value of 0.25 or less in the bivariable analysis were selected as candidate variables for multivariable logistic regression analysis. Multivariable logistic regression analysis was used to consider all these factors simultaneously.

The multivariable logistic regression analyses showed that participants who had a family history of epilepsy were 13.71 times more likely to develop epilepsy than those who did not have it (AOR = 13.71 [95% CI 3.032–22.006], *P* = 0.001). Participants with a history of febrile seizures were 14.57 times more likely to have epilepsy than those who did not have seizures (AOR = 14.57 [95% CI: 2.93–24.52], *P* = 0.001). Those who sustained head injuries were 6.85 times more likely to have epilepsy than those who did not (AOR = 6.85 [95% CI 1.779–16.402], *P* = 0.005). Those who used latrines were 0.028 less likely to develop epilepsy than those who did not; AOR = 0.028 (95% CI 0.008–0.043) *P* = 0.000 (Table 4).

**Table 4:** Determinants of epilepsy using multivariable logistic regression analysis among study participants in public health institutions in Dawo district, 2023.

**Explanatory variable**	**Category**	**Case No (%)**	**Control No (%)**	**COR (95% CI)**	**AOR (95% CI)**	***P*-value**
Family history	Yes	19 (31.2)	6 (5)	8.59 (3.2–2.98)	13.71 (3.03–22.00)	0.001^*^
	No	42 (68.8)	114 (95)	1	1	
Age	0–19	16 (26.2)	14 (11.7)	1.52 (0.59–3.91)	4.87 (0.66–15.74)	0.077
	20–39	27 (44.3)	82 (68.3)	0.43 (0.02–1.34)	3.49 (0.05–4.76)	0.119
	Above 40	18 (29.5)	24 (20)	1	1	
Cerebral malaria	Yes	9 (14.75)	5 (4.2)	3.98 (1.27–12.46)	2.56 (0.36–17.96)	0.343
	No	52 (85.25)	115 (95.8)	1	1	
Febrile seizure	Yes	13 (21.3)	7 (5.8)	4.37 (1.64–11.63)	14.57 (2.93–24.52)	0.001^*^
	No	48 (78.7)	113 (94.2)	1	1	
History of drinking alcohol	Every day	3 (4.9)	2 (1.7)	7.2 (1.06–48.84)	0.14 (0.006–3.1)	0.216
	Occasional	48 (78.7)	70 (58.3)	3.29 (0.35–13.58)	0.02 (0.005–2.94)	0.088
	None	10 (16.4)	48 (40)	1		
Head injury	Yes	23 (37.7)	15 (12.5)	4.23 (2–8.95)	6.85 (1.78–16.40)	0.005^*^
	No	38 (62.3)	105 (87.5)	1	1	
Latrine	Yes	6 (9.8)	94 (78.3)	0.03 (0.012–0.07)	0.028 (0.008–0.04)	0.000^*^
	No	55 (90.2)	26 (21.7)	1	1	

^*^Show significant association.

## Discussion

This study aimed to assess the determinants of epilepsy in public health institutions in Dawo. The study evaluated factors such as family history of epilepsy, a family history of febrile seizures, head injuries, and the use of latrines as determinants of epilepsy.

The findings of this study reveal that having a family history of epilepsy increases the chance of acquiring epilepsy by 13.71 times more than their counterpart. This finding was consistent with an earlier study conducted in Cameron (19), Kenya (20), Athens Greece (13), and Egypt (21). This could be due to hereditary factors that predispose members of a family to epilepsy. In contrast to these findings, in studies conducted at Korea University Ansan Hospital, a family history of epilepsy was not associated with epilepsy (22). This might be due to differences observed in study participants as the study was conducted only on adult participants.

Another major finding is febrile seizures, which have significantly increased the risk of developing epilepsy by 14.57-fold, and this finding is consistent with those from studies conducted in Kenya (20), Egypt (21), Turkey, USA, India, and Brazil (16**?** –18). The discovery of a 14-fold increased risk of epilepsy following febrile seizures strongly suggests infections as a possible cause of epilepsy. The strong association observed with febrile seizures in this study, as well as in other studies, does not necessarily reflect a causal relationship but may mean that febrile convulsions represent an early expression of a low seizure threshold that later develops into epilepsy. In contrast to the finding of this study, a cross-sectional study conducted in children and adolescents (6–19 years) in a tertiary care hospital in India found no association between a history of febrile seizures and epilepsy (23). This might be due to differences in study design and study area.

Head injury is another major factor associated with a nearly 7-fold increased risk of developing epilepsy among those with a history of head injury. This finding is consistent with the studies conducted in Cameron (12), Egypt (21), USA (24), and Denmark (25). The increased risk may be attributed to the study areas being prone to traffic accidents, which are known to result in head injuries.

The availability of latrines was found to be a protective factor against epilepsy; participants who had latrines were 97.2% less likely to develop epilepsy than those who did not have toilets. This might be due to the availability of latrines having their own influence on the prevention of cysticercosis, which is transferred by the fecal-oral route and causes CNS infection (26). Additionally, having latrines is a sign of improved infrastructure, which may correlate with overall better living conditions, access to healthcare, and fewer environmental stressors that could contribute to neurological disorders. Improved public health and safety might indirectly reduce the risk of developing conditions such as epilepsy.

The history of cerebral malaria was not statistically different between the cases and controls in this study, which might be attributed to poor recall of events in the past. In contrast to these findings, a study conducted in Benin showed a significant association between cerebral malaria with epilepsy (14).

The history of regular alcohol drinking was not statistically different between the cases and controls in this study. In contrast to findings from studies conducted in Poland, regular alcohol consumption is significantly associated with epilepsy (14). This may be due to differences in study population.

The age category was not statistically different between the cases and controls in this study. In contrast to these findings, in studies conducted in the Republic of China, people aged 40–59 shared 1.8 times more risk of epilepsy than those aged 20–39 years (27). This could be due to differences in lifestyle and study design.

The marital status was not statistically different between the cases and controls in this study. In contrast to these findings, studies conducted in the Republic of China reveal people who never married had a 2.8-fold higher risk of epilepsy than those who married/lived with a partner (27). This could be due to cultural differences in marriage.

This study has both clinical and public health significance. The clinical importance of this study is in the assessment and management of patients with epilepsy by identifying specific risk factors associated with epilepsy in the Dawo District population. This knowledge can aid in early detection, diagnosis, and personalized treatment plans for individuals living with epilepsy. Healthcare providers can tailor their treatment approaches, counseling, and education programs based on the identified determinants. This personalized approach can enhance patient outcomes, treatment adherence, and overall quality of life. Understanding the determinants of epilepsy can also contribute to the development of effective preventive strategies. By identifying modifiable risk factors, healthcare professionals can implement different targeted interventions, such as health education campaigns, public awareness programs, and community-based initiatives to reduce the incidence and burden of epilepsy in the Dawo district population.

The public health significance of this study was the need for resource allocation. Epilepsy is a chronic condition that requires long-term management and access to specialized healthcare services. Through the identification of the specific determinants of epilepsy in the Dawo district, public health officials can allocate resources more efficiently. This can involve ensuring an adequate supply of antiepileptic drugs, establishing specialized epilepsy clinics or centers, and training healthcare providers to address the identified determinants effectively. Moreover, this study can help combat the stigma associated with epilepsy and promote a more inclusive society by emphasizing the need for accessible and equitable care for individuals living with epilepsy.

### Strength of the study

This study includes all public health institutions in the district, which increases its generalizability. Since the risk factors for epilepsy remain poorly understood, case-control studies provide a valuable approach for establishing associations with multiple potential risk factors.

### Limitations of the study

It was a facility-based study in which the study subjects came to the health facility for some health problems other than epilepsy and hence it may confound the true association. There is also the possibility of recall bias, which is one of the inherent limitations of a case-control study. Moreover, this study has limitations in assessing all possible risk factors for epilepsy, such as perinatal complications, neurodegenerative disease, and substance abuse. Therefore, going forward, a larger-scale study should be conducted.

## Conclusion

This study identified a family history of epilepsy, a history of febrile seizures, head injury, and the unavailability of latrine as strong independent predictors of epilepsy. The finding that adverse febrile seizures increased the likelihood of epilepsy shows that much of the epilepsy in Dawo district may be prevented by improved maternal, neonatal, and child care. It is recommended that the Dawo Health Office (DHO) at all levels, healthcare providers across all institutions, and relevant stakeholders collaborate on addressing this issue. The administration of the Dawo district should endeavor to reduce the incidence of head injury by stabilizing the peace of the community and identifying the major cause of head injury in the districts. The Woreda Health Bureau in Ethiopia should focus on the improvement of latrine availability and creating awareness for the community through health extension workers. Furthermore, this study recommends that future researchers conduct large-scale studies by including different variables that were not included in this study, such as perinatal complications, neurodegenerative disorders, substance abuse, and others.

## Data Availability

The raw data supporting the conclusions of this article will be made available by the authors, without undue reservation.
